# Potential Immunological Links Between Psoriasis and Cardiovascular Disease

**DOI:** 10.3389/fimmu.2018.01234

**Published:** 2018-06-01

**Authors:** Aparna P. Sajja, Aditya A. Joshi, Heather L. Teague, Amit K. Dey, Nehal N. Mehta

**Affiliations:** National Heart, Lung, and Blood Institute, National Institutes of Health, Bethesda, MD, United States

**Keywords:** psoriasis, cardiovascular disease, inflammation, atherosclerosis, vascular inflammation, inflammatory cytokines

## Abstract

Preclinical and clinical research provide strong evidence that chronic, systemic inflammation plays a key role in development and progression of atherosclerosis. Indeed, chronic inflammatory diseases, such as psoriasis, are associated with accelerated atherosclerosis and increased risk of cardiovascular events. Contemporary research has demonstrated plausible mechanistic links between immune cell dysfunction and cardiometabolic disease in psoriasis. In this review, we describe the role of potential common immunological mechanisms underlying both psoriasis and atherogenesis. We primarily discuss innate and adaptive immune cell subsets and their contributions to psoriatic disease and cardiovascular morbidity. Emerging efforts should focus on understanding the interplay among immune cells, adipose tissue, and various biomarkers of immune dysfunction to provide direction for future targeted therapy.

## Introduction

Inflammation is the hallmark of atherosclerosis (1). Preclinical and clinical research provide strong evidence that chronic inflammation is critical to the process of atherogenesis. Chronic inflammatory diseases, such as psoriasis, are associated with accelerated atherosclerosis and increased risk of cardiovascular events (2–6). Atherosclerosis is increasingly recognized as an inflammatory process, thus similarities between atherosclerosis and chronic, systemic inflammatory diseases have become an emerging focus of interest. Almost 20% patients with coronary heart disease lack conventional risk factors (7), supporting the importance of evaluating residual inflammatory risk (8). Chronic inflammatory diseases such as psoriasis have been shown to add 6% additional risk (9, 10) to the Framingham Risk Score (8, 9) highlighting the need to understand the role of immunological processes in cardiovascular disease (CVD) for better risk stratification and treatment strategies.

## Chronic Inflammation and Cardiovascular Co-Morbidities

Patients with chronic inflammatory diseases are predisposed to cardiometabolic diseases including obesity, hypertension, and dyslipidemia (11–16)—chronic inflammatory conditions common in the general population (17–19). Obesity, particularly visceral, is strongly associated with dysregulated expression of inflammatory cytokines such as tumor necrosis factor-alpha (TNF-α), interleukin-1 beta (IL-1β), and IL-6, as well as adiponectin and leptin, contributing to metabolic derangement and insulin resistance (13, 18, 20). Atherogenic metabolic dyslipidemia is common in chronic inflammation. Abnormalities include impaired reverse cholesterol transport ability of the HDL, increased LDL particle number, and decreased LDL size (21–23). Animal and human models have demonstrated innate immunity as well as experimental *in vivo* induction of inflammation *via* bolus of an inflammatory cytokine such as TNF-α or IL-6, results in release of adipokines and generation of peripheral insulin resistance (24–27). Moreover, anti-inflammatory therapies such as aspirin, colchicine, and more recently canakinumab have been effective in CVD treatment, supporting the critical role of inflammation in CVD (28–32).

One of the most common co-morbid conditions associated with psoriasis is psoriatic arthritis (PsA). Epidemiological data indicate that almost one-third patients with psoriasis also have prevalent PsA (33). Similar to psoriasis, PsA is associated with increased prevalence of traditional cardiovascular risk factors, greater subclinical CVD assessed as vascular inflammation (VI) by 18-FDG PET/CT and ultrasound-guided carotid plaque assessment and intima-media thickness measurement, and elevated rates of major adverse cardiovascular events (MACEs) (34–38). Furthermore, like psoriasis, traditional risk factors do not fully capture the risk of CVD in PsA (39, 40).

Recently, there is growing focus on shared immunological links between atherosclerosis and several other autoimmune diseases such as systemic lupus erythematosus, inflammatory bowel disease, human immunodeficiency virus infection, rheumatoid arthritis, and psoriasis. These all carry an accelerated CVD risk, thought to be partly attributable to inflammation-driven endothelial dysfunction, lipoprotein derangement, and metabolic dysfunction stemming from chronic inflammation (41, 42). In order to speed understanding of inflammatory cardiometabolic dysfunction, psoriasis has been utilized as a human model (3) to understand the role of innate and adaptive immunity in subclinical CVD (43, 44). The clinical implications of understanding how the inflammatory processes in psoriasis contribute to cardiovascular morbidity are vast since approximately 3% of the US population has psoriasis. Furthermore, observational reports have suggested that anti-inflammatory therapies commonly used to treat psoriasis may associate with reduced cardiovascular risk (45, 46).

## Potential Immunologic Links Between Psoriasis and CVD

### Psoriasis Is Associated With Subclinical and Clinical Atherosclerosis

In the last decade, multiple studies have demonstrated an association between psoriasis and both subclinical and clinical atherosclerosis, such as VI by ^18^F-FDG PET/CT, coronary artery calcium and non-calcified coronary plaque burden by coronary computed tomography angiography (44, 47–51). Population-based studies provide evidence of early subclinical and clinical CVD in psoriasis (2, 4, 52, 53). Research into the concept of psoriatic march (54) has led to an understanding of common cellular and molecular level links between psoriasis and atherosclerosis (55).

### Common Immune Cells Between Psoriasis and Atherosclerosis

#### T Cells

Studies in the last two decades have established psoriasis primarily as a T-cell-mediated disorder (56–60). While initial evidence implicated a predominant role of helper T cells type 1 (Th1) through downstream activation of macrophages, neutrophils, and CD8^+^ cytotoxic T lymphocytes (61), recent research shows the importance of the Th17 and other IL-17 producing cell types such as CD8^+^ T cells and γδ T cells (62). Although Th1 subtype is the most studied cell-type in psoriasis, different stages of this chronic inflammatory disease employ various cells of innate and adaptive immunity (62). All the subtypes of T cells involved in pathogenesis of psoriasis are also involved in atherosclerosis (63).

#### Th1 Cells—Helper T Cells Type 1

Activation of the innate immune system is the key event in beginning the inflammatory cascade in psoriasis. It primarily includes differentiation of T cells into Th1 cells catalyzed by IL-12 (62). Mechanistic studies in patients with psoriasis have suggested a preference of hematopoietic progenitors toward Th1 subtype (64). Th1 cells induce psoriatic inflammation by activating neutrophils, macrophages, and CD8^+^ cytotoxic T lymphocytes (61). Primary mediators of Th1 activity are interferon-gamma (IFN-γ), IL-2, and TNF-α, which act on keratinocytes and induce antimicrobial peptide production that subsequently continues the inflammatory cascade. Th1 cells are also critical to the process of atherosclerosis, a process thought to be primarily driven by IFN-γ, the hallmark cytokine of the Th1 response (65). In patients with unstable angina and acute coronary syndrome (ACS), Th1 cells were found to be elevated (66, 67). Furthermore, mechanistic studies have also established the role of IL-12 in the development and progression of early atherosclerotic plaques (68–70). In addition, IL-18, a Th1-promoting cytokine, has also been shown to have a role in atherosclerosis (71, 72). Finally, targeting Th1 differentiating transcription factor is shown to associate with reduced atherosclerotic plaques (73). An IL-12 stimulated activation of Th1 response with downstream release of pro-inflammatory cytokines is a common feature between psoriasis and atherosclerosis and is thought to contribute to subsequent endothelial dysfunction and T cell recruitment to the sites of atherosclerotic plaques (74). While the role of Th1 cells is profoundly studied, the function of Th2 cells remains a topic of controversy as multiple studies exist that support pro-atherosclerotic (75), atherosclerosis protective (76), and also null effect (77) of Th2 cells.

#### Th17 Cells—Helper T Cells Type 17

Th17 cells in psoriasis release different cytokines such as IL-17, IL-22, and TNF-α (78) and are also involved in macrophage-dependent and -independent stimulation of dendritic cells (DCs) to propagate the inflammatory response (79). They may be involved in increased production of angiogenic inflammatory mediators such as monocyte chemoattractant protein (MCP-1), nitric oxide, and vascular endothelial growth factor (80, 81). Similar to Th2 helper cells, there is conflicting data on the role of Th17 cells in atherosclerosis (82). Patients with ACS show increased Th17 cells and IL-17 compared with those with stable angina or non-cardiac chest pain (83, 84). There is mixed evidence from mechanistic models: with some mouse models supporting the pro-atherogenic role of Th17 and IL-17 (85–87), while others have found low IL-17 mRNA in atherosclerotic plaques and overall attenuated disease development with high prevalence of Th17 cells (88–90). We later discuss the emerging role of neutrophils in the IL-17 axis, a possible mechanistic link; however, further clinical and translational research is necessary to elucidate the differential roles of Th17 and neutrophils in this pathway.

#### Regulatory T Cells (Treg Cells)

Regulatory T cells are a subset of T lymphocytes with a primary function to inhibit T cell activation and proliferation, through both cell-contact-dependent and cell-contact-independent anti-inflammatory cytokine (mainly TGFβ and IL-10) driven mechanisms (91). Treg inhibitory function is distinctly impaired in psoriasis (92, 93), contributing to the chronic auto- inflammation in psoriasis. ACS patients are also known to have decreased levels of circulating Treg cells with reduced efficacy and increased apoptosis susceptibility (94–97). Treg cells play an anti-inflammatory role in atherosclerosis through endothelial cell modulation, plaque stabilization by decreasing macrophages and lipid content and increasing smooth muscle cell and collagen, inhibition of pro-inflammatory cytokines, and secretion of anti-inflammatory cytokines such as TGFβ, IL-10, and IL-35 (91). Identification of common targets to reverse Treg cell dysfunction or to augment their activity in psoriasis may represent treatment mechanisms for both psoriasis and atherosclerosis simultaneously.

Finally, there are several other T cell phenotypes that have been identified in psoriasis skin lesions, such as CD4^+^, CD8^+^ T cells, CD146^+^, and γδ T cells (98). However, their role in psoriasis and atherosclerosis need to be further explored. While the traditional paradigm of T cell lineages might predominate shared mechanistic links between psoriasis and atherosclerosis, there is significant heterogeneity and plasticity within the T cell subtypes. T cell predominance may change in context of subtype preponderance with the natural disease course, specifically, a switch from Th1 dominated profile in early initiation phase of psoriasis to a Th17 governed response in the chronic inflammatory phase with both involved in atherosclerosis progression (99).

#### Dendritic Cells

In psoriasis, DCs not only act as antigen presenters and cytokine producers but also play an important part of bridging the innate and adaptive immune systems in continuing the chronic inflammation inducing cascade (43, 79). While pDCs are important in initiation of psoriasis *via* type 1 IFN responses (62, 100), mDCs are key mediators for specific Th cell expansion *via* IL-12 and IL-23 secretion (79). While new evidence suggests a role for DCs in atherosclerotic plaque build-ups, plaque vulnerability through cholesterol metabolism and adaptive immune response modulation (101), their shared role in psoriasis and atherosclerosis needs further research.

#### Monocytes and Macrophages

Monocytes and macrophages are cellular hallmark of atherosclerosis (1) and are also involved in pathogenesis of psoriasis (102). While macrophages are traditionally subclassified as pro-inflammatory (M1) and anti-inflammatory (M2), they are known to be plastic and adapt to the surrounding milieu according to the underlying pathological state (103, 104). Furthermore, a preclinical *in vivo* and *in vitro* study demonstrated that chronic skin inflammation in psoriasis polarizes them toward the pro-atherosclerotic phenotype (99). These cells are involved in ACS, and their increased expression and activity is also present in vulnerable plaques (105). Novel evidence has recently suggested that a complex interplay involving neutrophil–macrophage cross-talk is crucial to the process of atherosclerosis and ACS (106–108). As these cells are involved throughout the process of atherosclerosis from plaque development to complications, such as ACS, and also play a significant role in psoriasis, further research may provide new avenues for treatment of both these conditions.

#### Neutrophils

Despite being the most abundant white blood cell in the circulation, neutrophils have received little attention in the pathophysiology of atherosclerosis and psoriasis. Recent mouse models and clinical trials have demonstrated the mechanistic role of neutrophils in psoriasis and atherosclerosis through the IL-17 driven keratinocyte hyper-proliferation, leading to chronic skin inflammation (109, 110). Psoriasis patients are known to have higher serum levels of IL-17 compared with healthy controls; however, the paradigm of Th17 as the predominant cellular source of IL-17 in psoriatic lesions is no longer fully valid (111). Recent studies have demonstrated that cells of the innate immune system, such as neutrophils, mast cells, γδ T cells, and innate lymphoid cells, are the main sources of IL-17 in psoriasis. Furthermore, despite controversies, IL-17 is shown to have a role in atherosclerosis in clinical and mouse model-based studies (83–85, 87).

Psoriasis increases neutrophil activation and release of neutrophil-associated proteins. Proteins associated with neutrophils such as S100A8/A9 may further provide a link between psoriasis and cardiometabolic diseases (100). S100A8/A9 (MRP8/14) is released by activated neutrophils and upregulated in psoriatic lesional skin (100, 112). We demonstrated its strong association with both skin disease severity and VI (100). Collectively, evidence suggests that neutrophils and their proteins may contribute to the early atherosclerotic milieu in psoriasis and independently predict endothelial dysfunction.

A novel subtype of neutrophils, the low-density granulocytes (LDGs), are moving to the forefront of research in psoriasis and CVD pathophysiology. LDGs are characterized by high pro-inflammatory activity, altered phagocytic function, elevated type I interferon production, and high abundance in atherosclerotic plaques and plasma of psoriasis patients (113). At the gene expression level, LDGs differ from their autologous normal-density granulocytes (NDGs) counterparts, as well as from healthy control neutrophils (114–116). LDGs also differ phenotypically from NDGs. Of these differences, the most compelling is their enhanced capacity to spontaneously form neutrophil extracellular traps (NETs). This novel defense mechanism termed NETosis goes beyond classical phagocytosis, where NETs are formed as a result of release of cytosolic granule proteins bound to nuclear material catalyzed by peptidylarginine deiminase 4 (117). Although NETs are beneficial in antimicrobial defense, they may act as a source of autoantigens and are implicated in the development of autoimmune diseases especially psoriasis, as well as other diseases including systemic lupus erythematosus, atherosclerosis, preeclampsia, acute lung injury, deep vein thrombosis, and cancer-associated thrombosis (118–121). Cholesterol crystals are shown to trigger NETosis, further potentiating atherosclerosis by macrophage priming, Th17 activation, and immune cell recruitment in plaques (108). NETs are also shown through immunochemical stains to directly induce endothelial dysfunction and plaque rupture in human carotid plaque sections (122). NETs may be involved in the initial injury of the endothelium during atherogenesis, with recent evidence demonstrating the presence of neutrophils and NETs at sites of plaque rupture and endothelial cell erosion in human carotid plaques, features which we hypothesized would be evident in early atherosclerosis in psoriasis.

## Adipose Dysfunction in Psoriasis

**Figure 1:**
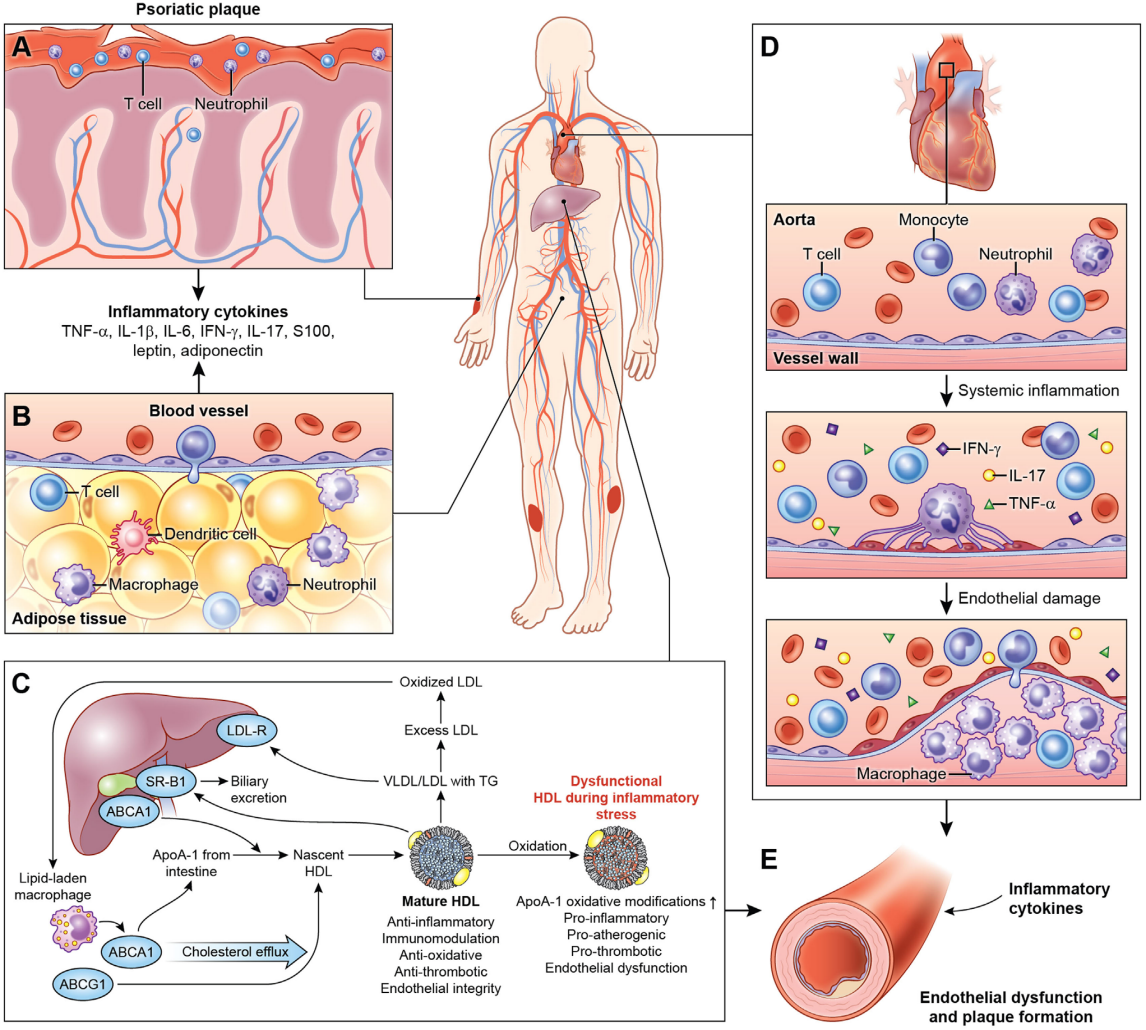
Systemic effects of chronic low-grade inflammation in psoriasis. **(A)** Psoriasis, both cutaneous and arthritic, is a low-grade chronic, systemic inflammatory disease associated with increased circulating pro-inflammatory cytokines. **(B)** Chronic inflammation in psoriasis is associated with adipose tissue dysfunction characterized by pro-inflammatory cytokines and adipokines associated with endothelial dysfunction. **(C)** Furthermore, psoriasis exhibits a deranged lipid profile and impaired HDL function, which in combination with chronic inflammation accelerate atherosclerotic vascular disease. **(D)** The vessel wall is infiltrated through a complex interplay of pro-inflammatory cellular components, cholesterol crystals, and various lipoproteins. Over the time, with build-up of the plaque, this atherosclerotic lesion poses a significant threat to blood flow and is prone to rupture, often accelerated by inflammation leading to myocardial infarction. **(E)** Thus, psoriasis and psoriatic arthritis upregulate T-cell, neutrophil chemotaxis, and keratinocyte activation and endothelial dysfunction leading to increased atherosclerosis in blood vessels. Abbreviations: TNF-α, tumor necrosis factor-alpha; IL, interleukin; IFN-γ, interferon-gamma.

Systemic inflammation associated with psoriasis also contributes to inflammation of the adipose tissue (20), harboring components of the innate immune system (Figure 1) (63, 123). The physiological distinction between visceral and subcutaneous adiposity has been considered an important determinant in assessing CVD risk. Visceral adiposity is highly metabolically active, and its dysregulation can alter the immune cell and adipokine profile, exacerbating endothelial dysfunction. Visceral adiposity is associated with subclinical CVD measured as VI by ^18^F-FDG PET/CT independent of cardiovascular risk factors in psoriasis (124). Furthermore, a decrease in visceral adiposity associated with an improvement of VI following 1 year of biologic anti-inflammatory therapy.

Psoriatic adipose tissue contains immune cells that influence cardiometabolic disease (20). T cells, B cells, DCs, neutrophils, mast cells, and adipose tissue macrophages (ATM) may contribute to obesity and insulin resistance, while eosinophils and Treg may protect against insulin resistance. ATM represent unique functional subset in psoriasis that are predisposed toward pro-inflammatory cytokine expression and adipose dysfunction, extending beyond the M1/M2 macrophage paradigm (20, 125, 126).

While visceral abdominal adiposity is being increasingly studied, there is emerging research that a local type of visceral adipose tissue, known as perivascular adipose tissue (PVAT), which surrounds most blood vessels (coronary arteries, the aorta, and microcirculation of the mesentery), may contribute to cardiometabolic disease (127, 128). Its anatomic proximity to the vasculature has led to research investigating the mechanisms of dysfunctional PVAT driven immune-mediated cross-talk in endothelial and vascular function under inflammatory conditions (127, 128). Mechanistic studies have demonstrated significant adipokine and chemokine (MCP-1, IL-8) production by PVAT and its ability to stimulate chemotaxis, contributing to progression of atherosclerosis (129, 130). Multiple pathways have been identified through which adipokines are implicated in CVD development—from direct vascular effects on endothelial function and smooth muscle migration to immune cell migration into the vascular wall through a potential “outside-in” inflammatory cascade (127). Recent efforts have led to a novel approach to image the PVAT and showed that it is associated with coronary inflammation in a dynamic fashion (131), with potential for prospective risk stratification.

Leptin is shown to be elevated in patients with psoriasis, to correlate with psoriasis disease severity and with indices of subclinical atherosclerosis (132, 133). We have previously exhibited an association between enhanced leptin and resistin activity with attenuated adiponectin activity in innate immune activation (24). Increased leptin and resistin promote expression of pro-inflammatory cytokines including TNF-α, IL-2, IL-6, and MCP-1, all of which are prothrombotic and drive VI through monocyte migration and macrophage activation (134). Finally, adipokines may contribute to the effect of insulin on the vasculature by contributing to changes in capillary recruitment (127).

Peri- and epicardial fat tissue are additional sources of visceral fat deposition, and a rich source of inflammatory cytokines that are associated with both subclinical and clinical coronary heart disease (128). Epicardial fat tissue has been reported to be significantly increased in psoriasis patients and may represent an independent risk factor for atherosclerosis (135).

## Biologic Therapies

The current generation of biologic agents target cytokines critical to the pathogenesis of psoriasis, including the three known major drivers: TNF-α, IL-23, and IL-17. The majority of most effective psoriasis treatments target the IL-23/Th17 pathway. These medications include the anti-IL-17 and anti-IL23p19 agents (Table 1). However, as novel therapies emerge, even today, anti-TNF agents remain the standard of care in general clinical practice (43, 136). While observational data in large payer-based or veterans association-based cohorts suggest a reduced risk for MACEs primarily with anti-TNF agents, no trials assessing direct cardiovascular effects of these medications in psoriasis patients exist to date (137–140). Although effective in treating psoriasis, interestingly, these therapies have been proven of no use in rheumatoid arthritis, another chronic inflammatory disease where the IL-23/Th17 axis plays an important role. The rationale behind these contradictory findings in two major inflammatory diseases currently remains unclear (141, 142).

**Table 1:** Biologic treatment options to treat psoriasis.

Biologic drug	Target cytokine	Cardiovascular effects
Etanercept	Tumor necrosis factor-α	Observational data indicating better CV outcomes. RCT for subclinical cardiovascular disease (CVD) demonstrating promising results. RCT dedicated for CV events not available (139, 140, 143)
Infliximab		
Adalimumab		

Secukinumab	Interleukin-17A and interleukin-17A receptor for brodalumab	Dedicated RCT for CV events unavailable
Ixekizumab		
Bimekizumab		
Brodalumab^a^		

Ustekinumab	Interleukin-12/23p40	RCT for subclinical CVD demonstrating favorable results. Dedicated RCT unavailable (144)
Briakinumab^a^		

Guselkumab	Interleukin-23p19	No data available yet for CV effects
Tildrakizumab		
Risankizumab		

Fezakinumab	Interleukin-22	Drug still in early development phase

*^a^Discontinued medications from the market*.

## Future Directions

Over the last decade, remarkable progress has been made for the treatment of moderate-to-severe psoriasis, especially with the advent of biologic therapies, which target specific cytokines, immune cells, and pathways. Moreover, the recent success of CANTOS (32) has demonstrated that inflammation reduction through direct IL-1β inhibition using a monoclonal antibody, canakinumab, in the absence of lipid lowering, can reduce CV event rates. As such, the emerging field of biologic treatments is exciting as it may provide therapeutic utility in psoriasis with added benefits of modulating CVD risk. Furthermore, completed and ongoing trials assessing the subclinical CVD in psoriasis have demonstrated promising findings (143, 144).

Finally, future research should focus on examination of complex inter-relationships between various conventional and non-conventional, inflammatory and non-inflammatory pathways to understand the heightened risk of CVD in disease conditions with underlying chronic inflammation.

## Conclusion

Increasing evidence demonstrates an important role of immune dysfunction linking psoriasis to cardiometabolic diseases including atherosclerosis. Future efforts in patients with chronic inflammatory disease like psoriasis should focus on elucidating the complex interplay among immune cells, adipose tissue, and various biomarkers of immune dysfunction. The shared mechanistic links between psoriasis and atherosclerosis provide promising avenues in targeted treatment for both diseases, especially in light of the recent trial CANTOS (32), which demonstrated reduced incidence of recurrent cardiovascular events after treating residual inflammation in patients with known coronary artery disease.

## Author Contributions

AS and NM conceived and designed research. AS, AJ, HT, AD, and NM contributed to both manuscript writing and critical review.

## Conflict of Interest Statement

NM is a full-time US Government Employee and receives research grants to the NHLBI from AbbVie, Janssen, Celgene, and Novartis. All other authors have nothing to disclose.
